# Effect of High-Dose Oral Rabeprazole on Recurrent Bleeding after Endoscopic Treatment of Bleeding Peptic Ulcers

**DOI:** 10.1155/2012/317125

**Published:** 2012-09-25

**Authors:** Hyung-Keun Kim, Jin-Soo Kim, Tae-Ho Kim, Chang-Whan Kim, Young-Seok Cho, Sung-Soo Kim, Hiun-Suk Chae, Sok-Won Han, Yong-Wan Park, Hye-Suk Son, Jeong-Yo Min, Guen-Jong Cho, Jung-Sun Bag, Son-Ook Choi

**Affiliations:** ^1^Division of Gastroenterology, Department of Internal Medicine, The Catholic University of Korea College of Medicine, Uijeongbu St. Mary's Hospital, 65-1 Geumo-dong, Kyunggi-do, Uijeongbu City 480-717, Republic of Korea; ^2^Division of Gastroenterology, Department of Internal Medicine, The Catholic University of Korea College of Medicine, Seoul St. Mary's Hospital, 505 Banpo-dong, Seocho-gu, Seoul 137-701, Republic of Korea; ^3^Division of Gastroenterology, Department of Internal Medicine, The Catholic University of Korea College of Medicine, Bucheon St. Mary's Hospital, 327 Sosaro, Wonmi-gu, Bucheon 420-717, Republic of Korea

## Abstract

*Background*. The aim of this study was to compare the effect of high-dose oral rabeprazole versus high-dose IV PPI on rebleeding after endoscopic treatment of bleeding peptic ulcers. *Methods*. This was a two-center, prospective, randomized, controlled trial. Patients with a high-risk bleeding peptic ulcer had endoscopic hemostasis and were randomly assigned to the high-dose oral rabeprazole group (20 mg twice daily for 72 hours) or the high-dose IV omeprazole group (80 mg as a bolus injection followed by continuous infusion at 8 mg/h for 72 hours). *Results*. The study was stopped because of slow enrollment (total *n* = 106). The rebleeding rates within 3 days were 3.7% (2 of 54 patients) given oral rabeprazole and 1.9% (1 of 52 patients) given IV omeprazole (*P* = 1.000). The rebleeding rates after 3 days were 1.9% and 0% (*P* = 1.000), respectively. The surgical intervention rates were 3.7% and 0% (*P* = 0.495), and the mortality rates were 1.9% and 0% (*P* = 1.000), respectively. *Conclusions*. The effect of high-dose oral rabeprazole did not differ significantly from that of high-dose IV omeprazole on rebleeding, surgical intervention, or mortality after endoscopic treatment of bleeding peptic ulcers, but this requires further evaluation.

## 1. Introduction

A bleeding peptic ulcer is the most common cause of upper gastrointestinal bleeding (UGIB) [1, 2]. Although the incidence of UGIB has decreased slightly, the overall mortality rate from a bleeding peptic ulcer has remained around 5% to 10% for the past three decades [3–5].

About 25% to 30% of patients with bleeding peptic ulcers have major stigmata of ulcer hemorrhage, which is associated with a high-risk for rebleeding when treated by medical therapy alone [6, 7]. Recurrent hemorrhage is probably the most important predictor of death from UGIB [8] and influences other important end points such as the need for a transfusion or surgery and length of hospital stay. Endoscopic hemostatic therapy is the treatment of choice for patients with a high-risk bleeding peptic ulcer [9, 10]. However, although successful hemostasis can usually be obtained with endoscopic treatment, rebleeding occurs in 15% to 25% of high-risk patients [6, 11–13]. Acid suppression, especially using proton pump inhibitors (PPIs), reduces the risk of rebleeding following endoscopic hemostasis of a bleeding peptic ulcer [7, 14, 15]. Therefore, a combination of endoscopic and pharmacologic therapy is the current standard management for patients with bleeding peptic ulcers, and high-dose intravenous (IV) PPI is recommended in patients who have undergone successful endoscopic therapy [16]. However, recent North American data suggest that the currently used high-dose IV PPI regimens may not achieve the target pH value of 6, regardless of whether the PPI is administrated orally or intravenously [17]. The optimal regimen and route for administration of PPIs remain controversial.

Previous Asian studies reported that the use of oral PPIs is effective under certain circumstances in the treatment of bleeding peptic ulcers [18–20]. However, no large-scale, prospective, randomized, controlled trials have compared the efficacy of oral versus IV PPI on recurrent bleeding after endoscopic treatment in patients with bleeding peptic ulcers. The aim of our prospective, randomized study was to compare and evaluate the effect of high-dose oral rabeprazole versus high-dose IV PPI on rebleeding after endoscopic treatment of bleeding peptic ulcers.

## 2. Methods

This was a two-center, prospective, randomized study to compare the effect of high-dose oral rabeprazole versus high-dose IV omeprazole on rebleeding after endoscopic treatment of bleeding peptic ulcers (ClinicalTrials.gov: NCT00838682). The study was conducted by investigators at Uijeongbu St. Mary's Hospital and Bucheon St. Mary's Hospital, The Catholic University of Korea, in accordance with the Declaration of Helsinki and the International Congress on Harmonisation Consolidated Guideline on Good Clinical Practice. The protocol was approved by the institutional review board of Uijeongbu St. Mary's Hospital and Bucheon St. Mary's Hospital. All subjects gave written informed consent and were enrolled from April 1, 2006, to April 19, 2007, and from October 1, 2007, to December 31, 2008.

### 2.1. Study Population

Patients who presented with overt or suspected UGIB based on hematemesis and/or melena were eligible. These eligible patients were required to have a peptic ulcer with active bleeding (Forrest classification Ia: spurting or Ib: oozing) or a nonbleeding lesion (IIa: nonbleeding visible vessel or IIb: adherent clot) on emergency endoscopy performed within 24 hours after hospitalization. Patients aged 16 years or older who achieved primary hemostasis with endoscopic hemostatic treatment were eligible. Exclusion criteria were refusal of the endoscopic procedure, complications from a peptic ulcer that required operative treatment such as gastric outlet obstruction or peptic ulcer perforation, serious concurrent disease such as malignant tumors or end-stage diseases, pregnancy, history of gastrectomy or vagotomy, severe hepatic disease, known hypersensitivity to proton pump inhibitors, age under 16 years, and epilepsy.

### 2.2. Study Design

Eligible patients who presented to the hospital because of overt or suspected UGIB and who were diagnosed with a bleeding peptic ulcer on emergency endoscopic finding were treated within 24 hours. Patients who achieved primary hemostasis with the endoscopic treatment were randomized into two groups using a random number table. All randomized patients were required to start a study drug within 24 hours of arrival at the emergency room. The IV omeprazole group received high-dose IV omeprazole for 72 hours, and the oral rabeprazole group received high-dose oral rabeprazole for 72 hours. Follow-up endoscopic examination was performed on days 1 and 3. If recurrent bleeding was suspected, repeat endoscopy was performed regardless of the prescheduled day. After the initial 72 hours, patients were discharged if they were stable without suspected rebleeding. Both groups received maintenance PPI therapy with rabeprazole 10 mg once daily from day 4 to week 6. The final follow-up endoscopic examination was performed in week 6.

### 2.3. Endoscopic Hemostasis

The modality of endoscopic hemostasis was either monotherapy or combination therapy using one of the following treatment modalities: epinephrine injection (1 : 10,000 diluted in saline), heater probe, monopolar or bipolar electrocoagulation, argon plasma coagulation, or hemoclip. The hemostatic method considered the most effective was selected by the investigators based on the patient's status, ulcer type, ulcer location, and bleeding pattern during the initial emergency endoscopy. The hemostatic treatment continued until active bleeding stopped and visible vessels disappeared. 

### 2.4. Study Medication

In the oral rabeprazole group, rabeprazole 20 mg twice daily for 72 hours was given orally. In the IV omeprazole group, omeprazole 80 mg was injected intravenously as a bolus followed by continuous infusion at 8 mg/h for 72 hours.

### 2.5. Outcomes

The primary end point was the occurrence of rebleeding within 3 days after the successful initial endoscopic hemostasis. Clinical rebleeding was defined as the reoccurrence of hematemesis following the resolution after the initial endoscopic hemostasis and redevelopment of shock following stabilization of vital signs. Clinical rebleeding was confirmed immediately with emergency endoscopy. The secondary end points included rebleeding after day 3, death due to rebleeding or any cause, the need for surgery, and cure of ulcer by week 6.

### 2.6. Safety

Adverse events were recorded beginning from the time the study patients signed the study consent form and included all adverse events encountered during the study. Adverse events included any change from the pretreatment condition including symptoms, physical findings, or laboratory values. 

### 2.7. Sample Size Estimate

A previous trial [7] showed that the rate of rebleeding is about 5% when initial endoscopic hemostasis is followed by a high-dose IV omeprazole for the treatment of bleeding peptic ulcers. Thus, the effect of high-dose IV omeprazole on the prevention of rebleeding was expected to be 95%. The effect of high-dose oral rabeprazole on the prevention of rebleeding was postulated as 85%. If a difference in the effects between the two regimens was within 10%, the effect of high-dose oral rabeprazole would be regarded as being not inferior to that of high-dose IV omeprazole. Assuming a potential dropout rate of 10%, the sample size needed to detect a reduction of 10% in the rate of rebleeding in the noninferiority clinical study with an *α* error of 0.05 (one sided), a *β* error of 0.2, and a power of 0.8 was calculated as 124 patients for each treatment arm of the trial and a total of 248 patients.

### 2.8. Statistical Analysis

Student's *t* test was used to compare the numerical variables between the two groups. Pearson's *χ* ^2^ test and Fisher's exact test were used to compare the categorical variables. All statistical tests were two tailed and were analyzed using SPSS software version 11.0 (SPSS, Chicago, IL, USA). A *P* value <0.05 was considered significant.

### 2.9. Study Termination

Although the estimated sample size was not achieved, the study was terminated by the investigators because of slow enrollment. After termination of the study and completion of case report forms, the final analysis was performed. All results were analyzed on an intention-to-treat (ITT) basis.

## 3. Results

### 3.1. Demographics and Baseline Characteristics

**Figure 1:**
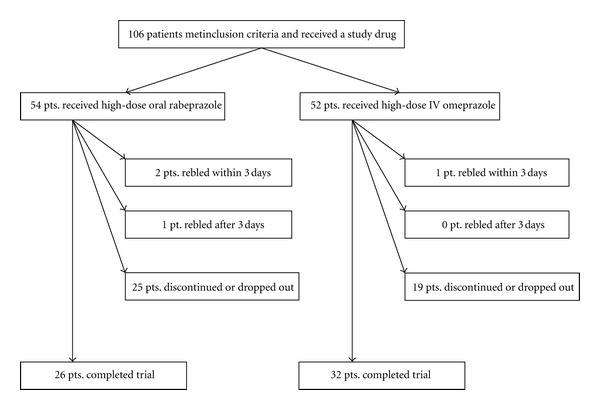
Flow chart. *For full details of patients who discontinued or dropped out, see Table 4.

A total of 106 patients, aged 19–87 years, with a bleeding peptic ulcer who met the inclusion criteria were enrolled in the study and received a treatment assignment. The ITT population comprised all 106 of the patients who received at least one dose of one of the study drugs; because all 106 patients received a study drug, none of the patients was excluded from the analysis. Fifty-four patients received high-dose oral rabeprazole, and 52 patients received high-dose IV omeprazole. Of the 106 patients, 62 patients completed the study, and 44 withdrew from the study (Figure 1). The patients' demographics and baseline characteristics did not differ between treatment groups (Tables 1 and 2). Few patients in either group took nonsteroidal anti-inflammatory drugs (NSAIDs) before hospitalization. 

### 3.2. Study Outcomes

 The results of ulcer rebleeding are shown in Table 3. The rebleeding rate within 3 days was 3.7% (2 of 54 patients) in the oral rabeprazole group and 1.9% (1 of 52 patients) in the IV omeprazole group (*P* = 1.000). All three patients with rebleeding within 3 days rebled on day 1. Of the two patients who experienced rebleeding in the oral rabeprazole group, one had a duodenal ulcer and the other had a duodenal ulcer with double pylorus; the patient who experienced rebleeding in the IV omeprazole group had a gastric ulcer. *H. pylori* status was unknown in the two patients in the oral rabeprazole group and positive in the patient in the IV omeprazole group. The stigmata of hemorrhage in the rebleeding patients involved the oozing and nonbleeding visible vessel type in the oral rabeprazole group and the adherent clot type in the IV omeprazole group. The rebleeding rate after 3 days was 1.9% (1 of 54 patients) in the oral rabeprazole group and 0% (0 of 52 patients) in the IV omeprazole group (*P* = 1.000). One patient with rebleeding after 3 days rebled at day 9. This patient had a duodenal ulcer, and the *H. pylori* status was positive. The stigmata of hemorrhage in this patient involved the nonbleeding visible vessel type. The overall rebleeding rate at 30 days was 5.6% (3 of 54 patients) in the oral rabeprazole group and 1.9% (1 of 52 patients) in the IV omeprazole group (*P* = 0.618). No additional rebleeding episodes occurred between 10 and 30 days. The mean number of transfusions of units of red blood cells and the duration of hospitalization were not significantly different between the oral rabeprazole and the IV omeprazole groups.

### 3.3. Detailed History of the Patients Who Underwent Surgery

 Two patients (3.7%) in the oral rabeprazole group and none in the IV omeprazole group underwent surgery (*P* = 0.495). One patient had a duodenal ulcer and rebled at day 1. This patient underwent emergency surgery because of failure to achieve hemostasis at the repeat endoscopy. The uncontrolled bleeding was caused by a submucosal tumor with a bleeding ulcer in the duodenal bulb adjacent to the pyloric ring in the surgical field, and the submucosal tumor was removed. The pathologic diagnosis of this submucosal tumor was heterotopic pancreas and adenomyoma in the duodenum, and this patient was excluded in the per-protocol analysis. The other patient also had a duodenal ulcer with double pylorus and rebled at day 1. The rebleeding in this patient was controlled at the repeat endoscopy, and this patient was then given the scheduled IV omeprazole because participation in the study had ended for this patient. However, this patient underwent pyloroplasty with truncal vagotomy because of failure to achieve hemostasis for the recurrent bleeding that occurred on day 8. This patient had diabetes mellitus and hypertension as comorbid diseases, and consumed alcohol and smoked regularly before hospitalization. This patient died on day 30; the cause of death was respiratory infection, surgical wound infection, and wound bleeding, which occurred after wound dehiscence. This patient was the only death in the oral rabeprazole group (1.9%), but the mortality rate did not differ significantly between treatment groups (*P* = 1.000).

### 3.4. Adverse Events

 There were few adverse events reported. Two adverse events in the oral rabeprazole group were elevation of hepatic enzyme levels and generalized tonic seizure-like activity. The elevated hepatic enzyme levels were resolved within several days. The seizure-like activity, possibly caused by hyperventilation, was resolved within 15–20 seconds by conservative management; no sequela was seen during the 6-week follow-up period. The two adverse events in the IV omeprazole group were elevation of hepatic enzyme levels and premature ventricular beats. The elevated hepatic enzyme levels were resolved within several days. The patient with premature ventricular beats had a history of percutaneous coronary angioplasty because of angina and had no concomitant symptom. No severe adverse events resulted from withdrawal from the study in either group.

### 3.5. Study Discontinuation

Table 4 shows the number of patients who withdrew after receiving at least one dose of the study medication and the primary reason for withdrawal. The most frequent reason for discontinuation was loss to follow-up at 6 weeks in both groups. The next reason for discontinuation was detection of one or more exclusion criteria after enrollment.

## 4. Discussion

The purpose of this prospective, randomized, controlled study was to compare the effect of high-dose oral rabeprazole versus high-dose IV PPI on rebleeding after endoscopic treatment of bleeding peptic ulcers with high-risk stigmata. Our study showed no significant differences in the effects of high-dose oral rabeprazole compared with high-dose IV omeprazole on rebleeding, the need for surgical intervention or blood transfusion, hospital stay duration, and mortality rate after endoscopic hemostasis in patients with a high-risk bleeding peptic ulcer. We note that our study had a small sample size.

This two-center trial was stopped by the investigators before the planned enrollment was completed. Recruitment in the study was slower than anticipated, and smaller than expected proportion of the patients with high-risk bleeding peptic ulcers qualified for the study. There are several reasons for this. First, the number of medical exclusions such as inpatient ulcer bleeding and recent PPI treatment decreased enrollment into this study. Second, the strict study design (e.g., starting a study drug within 24 hours of presentation to the emergency room) substantially reduced enrollment. Third, this trial was initiated by the investigators and not by the study sponsor.

High-dose IV PPI therapy in patients with a high-risk bleeding peptic ulcer who receive endoscopic hemostatic therapy reduces rebleeding, surgery, and mortality [21, 22]. However, it is not possible to draw conclusions about the efficacy of lower IV doses or high-dose oral PPI therapy, and the optimal dosage and route for administration of PPIs to prevent rebleeding after endoscopic hemostasis of a bleeding peptic ulcer remains controversial [21, 23, 24]. Oral PPIs effectively improved clinical outcomes in patients with a bleeding peptic ulcer in previous Asian studies [18–20]. However, these studies used a placebo as the control. Two recent studies directly compared the effect of an oral PPI with that of IV PPI in patients with a bleeding peptic ulcer [25, 26]. However, these two studies did not evaluate outcomes as the clinical end points but as the pharmacodynamic end points. A recent randomized controlled trial assessed the clinical outcomes of oral PPI versus IV PPI after endoscopic epinephrine injection in patients with a bleeding peptic ulcer [27]. In this study, oral rabeprazole (20 mg twice daily for 3 days) and regular-dose IV omeprazole (40 mg IV infusion every 12 hours for 3 days) were compared. Rebleeding up to 14 days occurred in 16.7% of patients in the oral rabeprazole group and in 15.4% of patients in the IV omeprazole group (*P* = 0.83). This study showed that oral rabeprazole and regular-dose IV omeprazole were equally effective in preventing rebleeding, but this was a single-center, prospective, randomized trial, and the control was the regular-dose IV PPI. No large-scale, prospective, randomized, controlled trials have directly compared the efficacy of high-dose oral PPI versus high-dose IV PPI therapy, and only one small-scale, randomized, controlled trial has been published on this comparison. A pilot study in patients with nonvariceal upper GI bleeding compared high-dose oral pantoprazole (80 mg twice daily for 3 days) with high-dose IV pantoprazole (80 mg IV bolus and 8 mg/h infusion for 3 days) [28]. Rebleeding within 30 days occurred in no patients in the oral pantoprazole group and in two patients (15%) in the IV pantoprazole group (*P* = 0.46). Other clinical outcomes such as mortality, blood transfusion, and duration of hospitalization did not differ between treatment groups. However, this randomized, controlled trial was underpowered because it was a pilot study of only 25 enrolled patients.

In our study, rebleeding rates in both the oral and IV PPI groups (3.7% versus 1.9% within 3 days and 5.6% versus 1.9% overall) were much lower than those of previous reported trials. There are several possible reasons for this difference. First, the method of endoscopic hemostasis in our study was not limited because we included any method that was successful in primary endoscopic hemostasis. Second, there was a very low prevalence of NSAID use in our study (5.7% of total enrolled patients), which was lower than in other studies on bleeding peptic ulcers. Third, our study enrolled Korean patients only. Previous studies showed that certain groups of Asian patients exhibit an increased pharmacodynamic effect of PPIs because of smaller parietal cell mass and a higher prevalence of either a slow-metabolizer phenotype for PPI or the presence of *H. pylori* infection [29]. 

Our study has several limitations. First, this was a two-center study and was stopped before enrollment was complete. The small sample size meant that the results of our study were statistically underpowered. Second, our study enrolled Korean patients only. Whether high-dose oral PPI therapy would have produced similar results in a Western population requires further investigation [21, 29]. Third, our study was designed to be open label, raising the issue of possible bias. However, assessment bias was negligible because the defined end points were all standardized and objective.

In conclusion, this two-center, prospective, randomized, controlled trial of patients with a bleeding peptic ulcer after endoscopic treatment has shown that the effect of high-dose oral rabeprazole did not differ significantly from that of high-dose IV omeprazole on rebleeding, the need for surgical intervention or blood transfusion, hospital stay duration, or mortality rate. Our data suggest that high-dose oral rabeprazole may be able to replace high-dose IV PPI as the treatment of choice after endoscopic hemostasis of bleeding peptic ulcers in certain circumstances. However, larger randomized, controlled trials to compare directly high-dose oral PPI with high-dose IV PPI are needed to document the efficacy of high-dose oral PPI in patients with a bleeding peptic ulcer.

## Figures and Tables

**Table 1:** Demographics (ITT population).

	Oral rabeprazole group (*n* = 54)	IV omeprazole group (*n* = 52)	*P* value
Age (yr)	56.1 ± 16.2	57.1 ± 15.8	0.729
Age group			0.496
<70 yr	43	38	
≥70 yr	11	14	
Sex (M/F)			0.227
Male	46	39	
Female	8	13	
Symptoms at presentation			0.356
Hematemesis	11	7	
Melena	27	27	
Hematochezia	1	1	
Hematemesis + melena	12	12	
Hematemesis + hematochezia	2	0	
Other	1	5	
Alcohol use	30	31	0.698
Smoking	25	24	1.000
NSAID use	5	1	0.206
Pulse (beats/min)	86.7 ± 13.8	90.7 ± 16.7	0.183
Blood pressure (mm Hg)			
Systolic	109.4 ± 19.2	116.0 ± 23.2	0.111
Diastolic	67.6 ± 13.1	68.8 ± 14.3	0.662
Hemoglobin (g/dL)	9.9 ± 2.5	9.6 ± 2.7	0.569
Hematocrit (%)	29.4 ± 7.3	28.5 ± 7.8	0.549
Comorbid illness	34	36	0.543

ITT: intention-to-treat; IV: intravenous; NSAID: nonsteroidal anti-inflammatory drug.

**Table 2:** Baseline characteristics (ITT population).

	Oral rabeprazole group (*n* = 54)	IV omeprazole group (*n* = 52)	*P* value
Ulcer size (mm)	13.7 ± 12.2	10.5 ± 11.1	0.173
Ulcer type			0.415
GU	30	27	
DU	21	20	
GU and DU	3	5	
*Helicobacter pylori*			0.653
Positive	22	19	
Negative	24	28	
Unknown	8	5	
Stigmata of hemorrhage			0.912
Active arterial spurting	6	4	
Oozing	15	14	
Nonbleeding visible vessel	21	23	
Adherent clot	12	11	
Endoscopic treatment			0.254
Epi + hemoclip	36	29	
Epi only	7	8	
Hemoclip only	6	5	
Epi + APC	2	5	
Epi + mono	0	2	
Epi + hemoclip + APC	2	0	
Epi + hemoclip + mono	1	0	
EBL	0	2	
APC	0	1	

ITT: intention-to-treat; IV: intravenous; GU: gastric ulcer; DU: duodenal ulcer; Epi: epinephrine injection (1 : 10,000 dilution in saline); APC: argon plasma coagulation; mono: monopolar coagulation; EBL: endoscopic band ligation.

**Table 3:** Outcomes in the study patients with a bleeding peptic ulcer (ITT population).

	Oral rabeprazole group (*n* = 54)	IV omeprazole group (*n* = 52)	*P* value
Rebleeding within 3 days	2	1	1.000
Rebleeding after 3 days	1	0	1.000
Total rebleeding	3	1	0.618
Surgery	2	0	0.495
Death	1	0	1.000
Blood transfusion (units)	1.3 ± 1.4	1.4 ± 1.3	0.582
Duration of hospitalization (days)	8.0 ± 5.6	6.7 ± 2.1	0.124

ITT: intention-to-treat; IV: intravenous.

**Table 4:** Number (%) of patients who discontinued the study grouped by primary reason (ITT population).

	Oral rabeprazole group (*n* = 54)	IV omeprazole group (*n* = 52)
Loss to followup at 6 weeks	21 (38.9%)	14 (26.9%)
Detection of exclusion criteria after enrollment*	4 (7.4%)	3 (5.8%)
Patient request	0	1 (1.9%)
Protocol violation	0	1 (1.9%)

Total	25 (46.3%)	19 (36.5%)

ITT: intention-to-treat; IV: intravenous.

*Detection of exclusion criteria after enrollment included lung cancer, stomach cancer at another site, bleeding of a gastric adenoma, Billroth II subtotal gastrectomy, and bleeding of a duodenal submucosal tumor.
